# Marine Algae as Source of Novel Antileishmanial Drugs: A Review

**DOI:** 10.3390/md15110323

**Published:** 2017-10-29

**Authors:** Lauve Rachel Tchokouaha Yamthe, Regina Appiah-Opong, Patrick Valere Tsouh Fokou, Nole Tsabang, Fabrice Fekam Boyom, Alexander Kwadwo Nyarko, Michael David Wilson

**Affiliations:** 1 Institute for Medical Research and Medicinal Plants Studies, Yaoundé 13033, Cameroon; tsabang2001@yahoo.fr; 2 Department of Parasitology, Noguchi Memorial Institute for Medical Research (NMIMR), College of Health Sciences (CHS), University of Ghana, Legon LG 581, Ghana; MWilson@noguchi.ug.edu.gh; 3 Antimicrobial and Biocontrol Agents Unit, University of Yaoundé 1, Yaoundé 812, Cameroon; ptsouh@gmail.com (P.V.T.F.); fabrice.boyom@fulbrightmail.org (F.F.B.); 4 Department of Clinical Pathology, NMIMR, CHS, University of Ghana, Legon LG 581, Ghana; rappiah-opong@ug.edu.gh (R.A.-O.); anyarko@noguchi.ug.edu.gh (A.K.N.); 5 Department of Pharmacology and Toxicology, School of Pharmacy, CHS, University of Ghana, Legon LG 43, Ghana

**Keywords:** leishmaniasis, marine organisms, marine algae, macroalgae, antileishmanial activity

## Abstract

Leishmaniasis is a vector-borne neglected tropical disease caused by protozoan parasites of the *Leishmania* genus and transmitted by the female *Phlebotomus* and *Lutzomyia* sand flies. The currently prescribed therapies still rely on pentavalent antimonials, pentamidine, paromomycin, liposomal amphotericin B, and miltefosine. However, their low efficacy, long-course treatment regimen, high toxicity, adverse side effects, induction of parasite resistance and high cost require the need for better drugs given that antileishmanial vaccines may not be available in the near future. Although most drugs are still derived from terrestrial sources, the interest in marine organisms as a potential source of promising novel bioactive natural agents has increased in recent years. About 28,000 compounds of marine origin have been isolated with hundreds of new chemical entities. Recent trends in drug research from natural resources indicated the high interest of aquatic eukaryotic photosynthetic organisms, marine algae in the search for new chemical entities given their broad spectrum and high bioactivities including antileishmanial potential. This current review describes prepared extracts and compounds from marine macroalgae along with their antileishmanial activity and provides prospective insights for antileishmanial drug discovery.

## 1. Introduction

Leishmaniasis is a vector-borne disease caused by protozoan kinetoplastid parasites of the genus *Leishmania*. The disease is transmitted through the bite of infected female phlebotomine sandflies of the genera *Phlebotomus* and *Lutzomyia* respectively in the Old World (Europe, Asia and Africa) and in the New World (America). *Leishmania* life cycle is dimorphic and heteroxene with an extracellular fusiform and flagellated promastigotes stage within the midgut of the sandfly, and a morphologicaly distinct intracellular amastigote stage within macrophages of a mammalian host [1,2]. It is worth noting that based on these two stages of *Leishmania* parasite, various models have been developed for drug susceptibility tests. The promastigote model is often used, however, this model does not necessarily reflect the physiological situation, as the disease-causing stage of the parasite, amastigote resides inside the host cells. Axenic amastigote model is used as an alternative to the labor-intensive intracellular amastigote model. However, several reports distinguish between axenic amastigotes and intracellular amastigotes both in terms of drug susceptibility and protein expression [3]. Leishmaniasis is a disease which is distributed worldwide in the tropics, subtropics, and the mediterranean basin and affect both humans and animals [3,4]. This neglected tropical disease represents a major public health problem in 98 endemic countries where it is responsible for approximately 2–4 million new cases and around 70,000 deaths per year [4]. Leishmaniasis presents two main clinical manifestations including cutaneous leishmaniasis (CL) affecting macrophages resident in the skin and visceral leishmaniasis (VL) affecting cells of the mononuclear phagocyte system of liver, spleen, bone marrow, lymph nodes and intestine [1]. VL is the most severe form of leishmaniasis with about 0.2–0.4 million cases each year in the world [1,5]. Besides, VL represents the second deadliest parasitic disease after malaria and the third-most common cause of morbidity after malaria and schistosomiasis [6]. *Leishmania* species, which cause VL include *L. donovani*, *L. infantum*, *L. martiniquensis* and *L. tropica* [1]. However, CL is the most common manifestation of the disease, with between 700,000 to 1.2 million new cases every year. It usually causes ulcers on the face, arms and legs. Furthermore, CL occurs in three differents forms, namely, localised cutaneous leishmaniasis, diffuse cutaneous leishmaniasis, and mucocutaneous leishmaniasis. Many *Leishmania* species are responsible for CL, including *L. mexicana*, *L. amazonensis*, *L. braziliensis*, *L. panamensis*, *L. guyanensis*, *L. aethiopica*, *L. venezuelensis*, *L. lainsoni*, *L. shawi*, *L. peruviana*, *L. naiffi*, *L. lindenbergi*, *L. infantum*, *L. martiniquensis* and *L. waltoni* in the New World and *L. tropica*, *L. aethiopica* and *L. major* in the Old World [1].

For the past 60 years, the first line-drugs of choice for leishmaniasis treatment have been pentavalent antimonials that include two formulations: sodium stibogluconate (pentostam) and meglumine antimoniate (glucantime) with comparable efficacy. However, emergence of resistance to these drugs added to toxic effects has been reported [7]. The second line-drug amphotericin B exhibits excellent activity against resistant parasites, but prolonged administration and adverse effects are major shortcomings to its use. The newer lipid formulations of amphotericin B are amphotericin B liquid complex, liposomal amphotericin B and amphotericin B colloidal dispersion. These new formulations allow for short-term treatment and provide excellent activity at low concentration with less toxicity. They are however extremely costly for poor patients in many affected countries [7,8,9,10,11,12]. Pentamidine, miltefosine, paromomycin, azoles and allopurinol are other drugs used against *Leishmania* parasites [6,13,14]. For most of these drugs, there are many drawbacks in terms of undesirable side effects, emergence of drug resistance, relapse after treatment or long-course of treatment and high cost [3,12,13,15]. In addition to the afore-mentioned limitations, the increased number of co-infection with HIV have challenged drug discovery research over the past two decades [3,4,8,9]. Ideally, the new therapeutic agents should have more innovative mechanisms of action, higher efficacy, affordability and shorter treatment duration, and less toxicity to improve compliance for the treatment and reduce the likelihood of emergence of resistance by the parasites.

Natural products are considered as rich and credible sources of compounds for drug discovery. At least one-third of the current top twenty drugs on the market are derived from a natural source, and approximately 50% of the marketed drugs are classified as naturally derived or designed on the basis of natural compounds [16,17].

Many studies worldwide have focused on the prospection of terrestrial plants for the discovery of effective compounds against the causative agents of leishmaniasis but little attention has been paid to the biological properties of marine organisms [18]. The interest in marine organisms as a potential source of promising novel bioactive natural agents has increased in recent times [19,20]. About 28,000 compounds have been isolated from marine organisms with hundreds of new chemical entities [21,22,23]. Natural products from marine algae are known for their potent and broad spectrum bioactivities including antimicrobial, antiviral, anti-helminthic, antituberculosis, antimycobacterial, antioxidative, anticoagulant, anti-inflammatory, antipyretic, analgesic, anticancer, insecticidal, antidiabetic, and antiprotozoan activities [21,24,25,26,27,28,29,30]. Moreover, the ability of marine algae to grow through mariculture and their short generation time make them sustainable sources of active ingredients. This is considered an environment-friendly strategic approach that overcomes problems associated with the overexploitation of marine resources and the use of destructive collection methods [21]. Despite this great potential, no attempt has been made to provide an overview of marine algae with leishmanicidal properties.

This review presents the antileishmanial activity of marine macroalgae and their phytochemicals and provides prospective insights for antileishmanial drug discovery.

## 2. Marine Algae

Marine algae are aquatic eukaryotic photosynthetic organisms, diversified in size, from microalgae to macroalgae [21,31,32].

Microalgae are a polyphyletic group of unicellular marine algae that constitute one of the major components of marine and freshwater phytoplankton. They are primary producers and a food source for other marine organisms [33]. There are at least 40,000 to 70,000 species belonging to three different groups which are diatoms, dinoflagellates and flagellates [21,34]. Microalgae are known to produce numerous useful natural products, but compared to macroalgae, they have attracted little attention in the search for novel anti-infective compounds [35] particulary against *Leishmania* parasite with only one report paper [30].

Marine macroalgae commonly known as seaweeds are macroscopic and multicellular. With an estimation of more than 30,000 species, they represent a considerable part of the marine environment. Seaweeds have been used as human food from 600 to 800 BC in China and other countries in Asia [36,37]. Based on their pigmentation, macroalgae are classified into three main phyla which include red seaweed (Rhodophyta), brown seaweed (Phaeophyta) and green seaweed (Chlorophyta) [38,39].

## 3. Current Status of Antileishmanial Drug Discovery from Marine Macroalgae

An overview of the findings reported so far on the search for *Leishmania* parasites inhibitors from macroalgae is presented in Table 1, including information on the macroalgae species, the type of extract, the *Leishmania* species and parasites forms. Algae with a determined IC_50_ value ≤100 µg/mL, or with a percentage of inhibition >50% were reported in Tables. Also found on Table 1 are the methods used for evaluation of the activities, and the activity parameters (IC_50_ -Extract concentration that inhibited the proliferation of parasites by 50%, CC_50_ -Extract concentration that inhibited the proliferation of normal mammalian cells by 50% or percentage of parasite inhibition).

The amount of research studies focusing on therapeutic properties of macroalgae has increased in recent years. However, only few antileishmanial secondary metabolites have been reported in 33 papers describing the antileishmanial activity of 151 marine macroalgae against different *Leishmania* parasites viz. *L. infantum*, *L. donovani*, *L. major*, *L. amazonensis*, *L. Mexicana* and *L. braziliensis* (Table 1 and Table 2). From the data assembled in Table 1 and Table 2, it appears that marine macroalgae could inhibit *Leishmania* parasites with IC_50_ values as low as 0.27 μg/mL [51]. This level of activity denotes the presence of highly potent natural products in these organisms. The following two sections highlight the macroalgae that were reported to have been investigated for antileishmanial activity.

### 3.1. Macroalgae with Antileishmanial Properties

It is estimated that there are more than 30,000 macroalgae identified around the world [38] out of which only 151 have been investigated against *Leishmania* parasites.

Extracts from 48 species of brown macroalgae (Phaeophyceae) have been screened against *Leishmania* parasites. Spavieri et al. [40] assessed the antileishmanial activity of isopropyl alcohol-chloroform/methanol extracts from 21 brown macroalgae and found that all the extracts showed antileishmanial activity against axenic *L. donovani* amastigotes with IC_50_ values ranging from 6.4 to 77.4 µg/ mL (Table 1). *B. bifurcata* and *H. siliquosa* extracts were the most active with IC_50_ values of 6.4 and 8.6 µg/mL, respectively. However, *B. bifurcata* and *H. siliquosa* extracts showed an extent of cytotoxic effects against the mammalian skeletal myoblasts, L6 cells with CC_50_ values of 32.7 and 45.0 μg/mL, respectively [40]. Hydroalcoholic and ethyl acetate extracts from eight brown algae were also assessed for their inhibitory activity against axenic *L. donovani* amastigotes [41]. Ethyl acetate extracts of *B. bifurcata*, *D. dichotoma* and *D. polypodioides* showed strong activities with IC_50_ values in the range 3.9 to 10.8 μg/mL. Nevertheless, they also exhibited cytotoxic profile on L6 cells with a CC_50_ value of 6.0 μg/mL, indicating poor selectivity of the extracts for the parasites. In another study, hexane, ether and chloroform extracts from *B. bifurcata*, showed moderate activities against *L. infantum* promastigotes with IC_50_ values ranging from 46.83 to 63.83 μg/mL and CC_50_ values below 41 μg/mL on Brine Shrimp larvae of *Artemia salina* [42]. These results also suggest that the extracts have poor selectivities towards the parasites. The phytochemistry of *B. bifurcata* has been extensively investigated and compounds such as sterols, polyphenols, diterpenes have been reported. Eleganolone was described as the major oxygenated diterpene [28,62,63,64,65,66]. The compounds present in the alga may be responsible for the antileishmanial activities. According to Vonthron-Sénécheau et al. [41], the cytotoxicity of *B. bifurcata* could be explained by the presence of terpenoids with cytotoxic activity.

Brown macroalgae of Dictyotaceae family, viz. *Canistrocarpus*, *Dictyota* and *Stypopodium* genera (Table 1 and Table 2) appeared to be among the most interesting Phaeophyceae. In fact, six out of the seven species from *Dictyota* genus (*D. caribaea*, *D. ciliolata*, *D. dichotoma*, *D. menstrualis*, *D. mertensii* and *D. pfaffii*) as well as *C. cervicornis* and *S. zonale* exhibited potent antileishmanial activities against promastigote, axenic amastigote and intracellular amastigote of *L. donovani* and *L. amazonensis* with IC_50_ values ranging from 0.27 to 81.4 μg/mL [40,41,46,47,48,49,50,51]. Extracts from three species showed interesting activity profiles, with IC_50_ values below 10 μg/mL and selectivity indices (ratio of CC_50_ value to IC_50_ value) greater than 20. Amongst these, ethylacetate/hexane extract from *S. zonale* exhibited the strongest activity against promastigotes and intracellular amastigotes of *L. amazonensis* with IC_50_ values of 0.75l and 0.27 μg/mL respectively and with respective CC_50_ values of 29.5 and >50 μg/mL on murine peritoneal macrophages [47,51]. Also, ethylacetate and hexane extracts of *D. menstrualis* showed potent activity with IC_50_ values ranging from 0.7 to 0.75 μg/mL and 0.61 μg/mL, respectively against promastigotes of *L. amazonensis* and CC_50_ > 18.2 μg/mL on murine peritoneal macrophages. Hexane extract of *D. ciliolata*, showed a strong antileishmanial activity with an IC_50_ value of 1.15 μg/mL on *L. amazonensis* promastigotes with CC_50_ value of 23.5 μg/mL [47]. This suggests a good selectivity of the extract since selectivity index is about 20. Besides, extracts from others brown macroalgae were also found to have antileishmanial activity, including *C. barbata*, *C. crinata*, *Lobophora variegata*, *Padina* sp., *S. muticum*, *S. natans*, *S. oligocystum*, *S. furcellata*, *T. turbinata* and *U. pinnatifida*. These extracts moderately inhibited the growth of *Leishmania* parasites with IC_50_ values ranging from 10.9 to 90.9 μg/mL or percentage of inhibition values ranging from 80.9% to 98.5% at the tested concentration (Table 1). The organic extract of *T. turbinata* that showed IC_50_ value of 10.9 μg/mL and a selectivity index of 70.41 is also of interest [46] and could further be investigated for its antileishmanial metabolites.

The most screened macroalgae phylum was Rhodophyta where 80 species were investigated. Extracts from many studied species of this phylum were shown to exhibit activity against cutaneous and visceral *Leishmania* parasites (Table 1 and Table 2). For instance, ethanolic extracts from *O. pinnatifida* (IC_50_ = 6.25 μg/mL), *M. afaqhusainii* (IC_50_ = 32.6 μg/mL), *G. corticata* (IC_50_ = 37.5 μg/mL), *S. hatei* (IC_50_ = 14.1 μg/mL), *S. indica* (IC_50_ = 59.6 μg/mL), *C. clavulatum* (IC_50_ = 57.89 μg/mL), *B. leptopoda* (IC_50_ = 60.81 μg/mL) and water extracts from *G. salicornia* (IC_50_ = 46.0 to 74.0 μg/mL) and *G. corticata* (IC_50_ = 38.0 to 65.0 μg/mL) inhibited the growth of promastigotes of cutaneous leishmaniasis agent, *L. major* [25,50,55] with. *O. pinnatifida* extract being the most promising inhibitor. Crude extracts from *B. fruticulosa*, *C. jubata*, *C. rubrum*, *C. virgatum*, *C. verticillata*, *C. ovatum*, *C. granifera*, *C. officinalis*, *C. ramosa*, *C. purpureum*, *D. pedicellata*, *D. incrassata*, *G. crinale*, *G. pulchellum*, *G. gracilis*, *G. verrucosa*, *H. incurvus*, *H. equisetifolius*, *J. rubens*, *F. lumbricalis*, *L. articulata*, *M. stellatus*, *O. hybrid*, *O. pinnatifida*, *P. cartilagineum*, *P. rotundus*, *P. linearis* [45,54] and ethanol and n-butanol fractions from *C. hornemanni* [58] were shown to be active against the visceral leishmaniasis causative agent *L. donovani* with IC_50_ values ranging from 16.76 to 85.6 μg/mL. Apart from the extracts from *C. officinalis* and *D. pedicellata* which showed cytotoxicity with CC_50_ values of 88.6 and 14.7 μg/mL respectively, all the other samples presented acceptable selectivity indices with CC_50_ values ranging from >90 μg/mL [45,54] to >200 μg/mL [58]. *Asparagopsis* species showed promising activities and might serve as starting point for future search of bioactive compounds against visceral leishmaniasis. In fact, dichloromethane and hexane extracts from *A. taxiformis*, ethanol-hexane:ethylacetate and ethanol-ethylacetate fractions from *A. taxiformis* and *A. armata* also showed significant effect on *L. donovani* promastigotes with IC_50_ values ranging from 10 to 20 μg/mL [52]. Additionally, ethanol extract of *A. taxiformis* was shown to be active against promastigotes and axenic amastigotes of *L. infantum* with IC_50_ values of 25 μg/mL and 9 μg/mL, respectively and acceptable safety profile on VERO and DH82 cell lines (CC_50_ > 90 μg/mL) [53]. These activities of *Asparagopsis* species could be attributed to the presence of volatile halogenated compounds (halomethanes, haloethers, haloacetals) which have been otherwise described as responsible for the antimicrobial properties of *A. armata* [67].

Besides, hexane (HO) and dichloromethane (DO) fractions and subfractions from *B. tenella* and aqueous extract from *B. triquetrum* showed antileishmanial activity against *L. amazonensis* promastigotes with IC_50_ values ranging from 1.5 to 78.6 μg/mL [56,57]. Among these, subfractions HO2 (IC_50_ = 1.5 μg/mL), HO3 (IC_50_ = 2.7 μg/mL), DO1 (IC_50_ = 4.4 μg/mL) and DO2 (IC_50_ = 4.3 μg/mL) were the most active [56]. Considering the activities of hexane (IC_50_ = 17.4 μg/mL) and dichloromethane (IC_50_ > 100 μg/mL) fractions of *B. tenella* as compared to subsequent subfractions, it is likely that chromatography may have concentrated the active principles in those subfractions or eventually reduce antagonistic interactions among molecules. These subfractions can be worked up to purify/characterized the antileishmanial molecules. Furthermore, crude extracts from the red macroalgae *Laurencia* complex (*L. aldingensis*, *L. dendroidea*, *L. microcladia*, *P. flagellifera* and *P. perforata*) showed great potential as antileishmanial drug sources (Table 1) and must be further explored. Reported findings indicate that extracts from these macroalgae have in vitro antileishmanial activity with IC_50_ values ranging from 16.3 to 97.2 μg/mL against the insect-stage promastigotes of *L. amazonensis* and *L. mexicana* and 8.7 to 34.5 μg/mL against the mammalian-stage amastigotes of *L. amazonensis* [46,59,60]. Moreover, they were globally not cytotoxic with CC_50_ value ≥1000 μg/mL. Extracts of *L. aldingensis*, *L. dendroidea* and *L. microcladia* exhibited the most interesting activity profile with IC_50_ values ranging from 8.7 to 30.1 μg/mL. Thus, these extracts may contain potential therapeutic agents, which must be further investigated. The antileishmanial potency of *Laurencia* genus could be attributed to the presence of bioactive sesquiterpenoids. In fact, more than 700 compounds mainly sesquiterpenoids and rearranged derivatives from *Laurencia* species have been shown to display a vast array of biological activities (antiviral, antibacterial, antimalarial) [68,69,70].

Extracts from few Chlorophyta marine macroalgae have been reported to have activity against leishmaniasis agents (Table 1). Indeed, from the investigation of 23 green macroalga species, acetone extract of *A. saldanhae* was active against the promastigotes (87.9% inhibition at 50 μg/mL) and intracellular amastigote (IC_50_ = 23.9 μg/mL) of *L. braziliensis* with CC_50_ value of 294.2 μg/mL on J774.G8 macrophage cell line [26]. This implies that the extract has good selectivity of 12.3 and can be suggested for chemical work-up to identify its bioactive principles. Also, crude extracts of *C. racemosa*, *C. rupestris*, *C. bursa*, *C. fragile*, *U. intestinalis* and *U. lactuca* significantly inhibited the growth of axenic *L. donovani* amastigotes in culture, with IC_50_ values ranging from 5.9 to 31.76 μg/mL and acceptable selectivity toward mammalian L6 cells (CC_50_ > 90 μg/mL). Amongst these extracts, *U. lactuca* and *U. intestinalis* extracts showed the highest antileishmanial activities [45,48,61]. The data suggest that green macroalgae from *Ulva* species could be valuable sources of antileishmanial compounds. Approximately 50 marine algae species from *Ulva* genus have been identified. *Ulva* species are known for their various sulphated polysaccharide (SP) compounds with ulvan as the most important one [71]. Extracts and SP from *U. lactuca* have been found to have antimicrobial, anticoagulant, antiprotozoal, antioxidant, antiperoxidative, antihyperlipidemic, antiviral, anticancer and hepatoprotective activities at low concentrations [72,73], making them good starting points for drug discovery against many diseases. Aqueous extract of *H. opuntia* showed activity on promastigotes and intracellular amastigotes of *L. amazonensis* with IC_50_ values of 83.5 μg/mL and 70.7 μg/mL respectively and CC_50_ value of 526.4 μg/mL on macrophages [57]. Bioguided fractionation should allow identifying bioactive compounds from this species.

Among the different extracts tested, the ethyl acetate extracts were globally the most active. Seven macroalgae were extracted with a non-polar solvent, ethyl acetate viz. *B. bifurcata*, *C. cervicornis*, *D. polypodioides*, *D. ciliolata*, *D. dichrotoma*, *D. menstrualis* and *D. carnosa* (Table 1). All the extracts strongly inhibited the growth of *Leishmania* with IC_50_ values ranging from 0.7 to 10.8 μg/mL.

### 3.2. Isolated Compounds from Marine Macroalgae Screened for Antileishmanial Properties

Table 2 below contains a summary of isolated compounds from marine macroalgae that were screened against *Leishmania* parasites.

**Figure 1:**
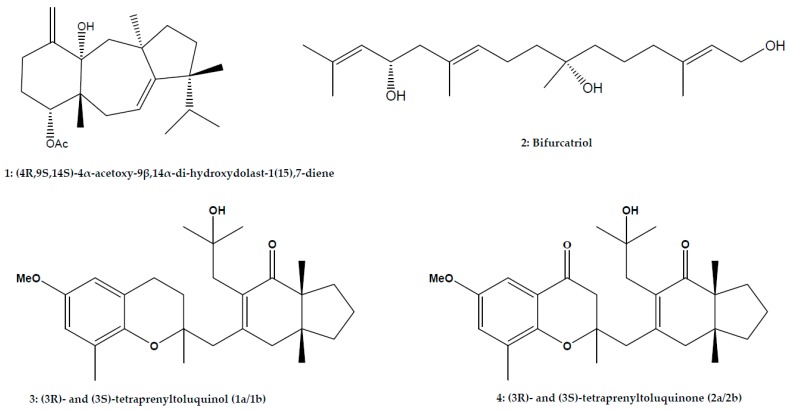

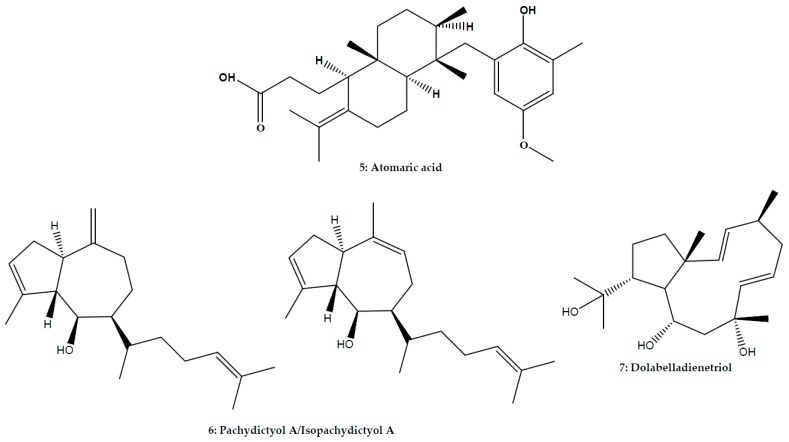

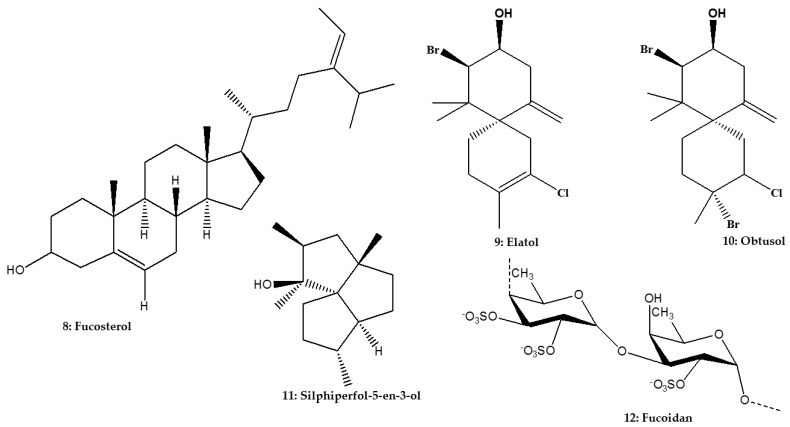
Structures of isolated compounds from marine macroalgae.

In the framework of the reported investigations of marine algae for antileishmanial drug search, isolated compounds (Figure 1) were screened against *Leishmania* parasites.

More than 50% of antileishmanial compounds isolated from macroalgae belong to the family of diterpenes (Prenylated guaiane, dolastane, secodolastane, xeniane, dolabellane, dichotomanes and meroterpenoids). These are largely distributed among the brown seaweeds of the Dictyotaceae family as major secondary metabolites with biological activities against a vast panel of pathogens, cancer cells and cardiovascular diseases [49,78,79,80,81].

From the diterpenes isolated, (4*R*,9*S*,14*S*)-4α-acetoxy-9β,14α-dihydroxydolast-1(15),7-diene (1, Figure 1), a 4-acetoxydolastane diterpene obtained from *Canistrocarpus cervicornis* showed interesting activity with IC_50_ values of 2.0 μg/mL, 12.0 μg/mL, and 4.0 μg/mL for promastigote, axenic amastigote and intracellular amastigote forms of *L. amazonensis*, respectively. Moreover, cytotoxicity assay showed that this compound was 93 times less toxic to the J774G8 macrophages than to *Leishmania* parasites. Studies on the mechanism of action revealed that the activity of this molecule may be due by its interference with the mitochondrial membrane potential and lipid peroxidation in parasite cells [43]. Compared to other isolated diterpenes, this compound was the most active, suggesting that the acetoxyl group in its structure seemed to increase the activity. On the other hand, the observed antileishmanial activity could be also related to the *Leishmania* species and the parasite stages, which often result in different drug susceptibility [3]. A new linear diterpene, bifurcatriol (2, Figure 1) isolated from *Bifurcaria bifurcata* was active against *L. donovani* with IC_50_ value of 18.8 μg/mL and CC_50_ value of 56.6 μg/mL on L6 rat myoblast cell line [74]. However, the assay was done on axenic amastigote form of the parasite, which is not the suitable target for antileishmanial drug discovery. Two meroditerpenoids, (3*R*)- and (3*S*)-tetraprenyltoluquinol (1a/1b) (3, Figure 1) and (*3R*)- and (3*S*)-tetraprenyltoluquinone (2a/2b) (4, Figure 1) isolated from *Cystoseira baccata* inhibited the growth of the promastigotes of visceral leishmaniasis parasite, *L. infantum* moderately with IC_50_ values of 44.9 μM and 94.4 μM, respectively [44]. (3*R*)- and (3*S*)-tetraprenyltoluquinol (1a/1b) also showed activity against the intracellular amastigotes with IC_50_ value of 25.0 μM and CC_50_ value of 126.6 μM on murine peritoneal macrophages. They were able to induce cytoplasmic vacuolization and the presence of coiled multilamellar structures in mitochondria as well as an intense disruption of the mitochondrial membrane potential. Comparing the structures of the two meroditerpenoids, it appears that the presence of a second carbonyl group in (3*S*)-tetraprenyltoluquinone (2a/2b) may be responsible for the observed decrease in activity. This assumption is reinforced by the lack of the second carbonyl group in the structure of another meroditerpenoid, atomaric acid (5, Figure 1) isolated from the active lipophilic extract of *Stypopodium zonale* [51]. In fact, atomaric acid showed a similar IC_50_ value 20.2 μM against the intracellular amastigote of *L. amazonensis.* This compound also inhibited promastigotes growth by up to 86% at 50 μΜ with low toxicity towards host cells (CC_50_ = 169.5 μΜ). The results showed that leishmanicidal activity of atomaric acid was independent of nitric oxide production, but the generation of reactive oxygen species may be at least partially responsible its activity against the amastigote form [51]. A mixture of diterpene isomers pachydictyol A/isopachydictyol A (6, Figure 1) isolated from the dichloromethane extract from *Dictyota menstrualis* showed antileishmanial activity against *L. amazonensis* promastigotes with IC_50_ value of 23.5 μg/mL. However, cytotoxic effect was detected on murine peritoneal macrophages (CC_50_ = 30.0 μg/mL) [47]. A dolabellane diterpene, Dolabelladienetriol (7, Figure 1), isolated from *Dictyota pfaffii* repressed the intracellular amastigote of *L. amazonensis* replication with IC_50_ value of 43.9 µM and a CC_50_ value >100 μM on murine peritoneal macrophages. At 100 μM, dolabelladienetriol inhibited 95.5% of promastigote growth [14]. Dolabelladienetriol was also active against *Leishmania*/HIV-1 co-infection with 56.0% inhibition at 50.0 μM. This compound was able to modulate macrophage activity by inhibiting nitrogen oxide and cytokines TGF-β and TNF-α production, which could be responsible of the activity of the compound.

One triterpene derivative, fucosterol (8, Figure 1) has been isolated from *Lessonia vadosa* a brown macroalga [75]. This phytosterol, was found to be significantly more active against the intracellular amastigotes of *L. amazonensis* and *L. infantum* (IC_50_ values of 7.89 μM and 10.30 μM respectively) compared to the vector stage, promastigotes (IC_50_ values of 55.0 μM and 45.0 μΜ, respectively) [75], indicating that the antileishmanial activity of fucosterol is dependent on a macrophage function. These results justify the intracellular amastigote model as the suitable model for drug-screening. In addition, fucosterol displayed little cytotoxicity against the host macrophagic cell line with CC_50_ value >100 μM [75].

With the aim to identifying the compounds responsible for the strong antileishmanial activity of *Laurencia dendroidea*, crude extract of the red macroalga was fractionated and sesquiterpene compounds, elatol (9, Figure 1), obtusol (10, Figure 1) and silphiperfol-5-en-3-ol (11, Figure 1) were isolated [26,60]. From the antileishmanial assay against the promastigotes and intracellular amastigotes of *L. amazonensis*, the two chamigrene sesquiterpne compounds (elatol and obtusol) were strongly active against the promastigote form (IC_50_ = 9.7 μg/mL and 6.2 μg/mL, respectively). Furthermore, they were strongly active against the intracellular amastigote form (IC_50_ = 4.5 μg/mL and 3.9 μg/mL) with low cytotoxicity. Althoough the triquinane sesquiterpene compound, silphiperfol-5-en-3-ol was also active, it was less active against both the promastigote (IC_50_ = 43.8 μg/mL) and the intracellular amastigote (IC_50_ = 48.7 μg/mL) forms. [26,60]. These similar IC_50_ values of elatol and obtusol is probably related to the presence of cyclohexane ring and chloride and bromine atoms that are not found in the triquinane sesquiterpene compound. None of these three sesquiterpnes significantly activated the production of nitric oxide by infected macrophages, suggesting that their antileishmanial activity is likely to be direct on the parasites rather than through macrophage activation [60]. Elatol induced the parasite’s killing through significant changes on parasite, including pronounced swelling of the mitochondrion, appearance of concentric membrane structures inside the organelle; destabilization of the plasma membrane and formation of autophagic vacuoles [26].

Among the sulfated polysaccharides screened, fucoidan (12, Figure 1), a polyanionic sulfated polysaccharide found in many brown algae was the most interesting. This compound showed an inhibitory effect on intracellular amastigote of *L. donovani* with 93% inhibition at 50.6 μg/mL. In vivo, a complete elimination of liver and spleen parasite burden was achieved at a dose of 200 mg/kg/day three times daily. Fucoidan was able to induce a protective response from the host by means of the production of cytokines and significant increment in the levels of reactive oxygen species and nitric oxide in infected macrophages, which may be involved in the observed reduction of the parasite multiplication [76]. Despite this promising potential, fucoidan, as a high-molecular-weight product has high hemorrhagic risk, poor solubility and bioavailability [82].

Overall, among the 151 macroalgae that were screened, only extracts from twelve species (*Botryoclada occidentalis*, *Canistrocarpus cervicornis*, *Caulerpa racemosa*, *Cystoseira baccata*, *Dictyota menstrualis*, *Dictyota pfaffii*, *Fucus vesiculosus*, *Gracilaria caudata*, *Laurencia dendroidea*, *Lessonia vadosa*, *Solieria filiformis* and *Stypopodium zonale*) were further investigated for identification of bioactive compounds (Figure 1). This denotes a gap in knowledge that should be filled in. Therefore, an integrated approach of identification of macroalgae with antileishmanial properties followed by identification of bioactive compounds should be undertaken to speed up the research and development of marine algae as sources of druggable molecules for treatment of Leishmania diseases.

## 4. Approaches Used for Assessment of Antileishmanial Activity by the Authors

Different approaches viz. microscopic, green fluorescent protein, resazurin, MTT, XTT, enzymatic hydrolysis of *p*-nitrophenyl phosphate and models viz. promastigotes, axenic amastigotes, intracellular amastigotes, and mouse model were used to determine the antileishmanial activity of natural products from marine algae.

In the microscopic assays, *Leishmania* spp. axenic amastigotes, promastigotes or harboring Green Fluorescent Protein (GFP) were treated with varying concentrations of natural products. After incubation, parasite viability was measured using microscopic counting technique [43,59]. In intracellular antileishmanial assay, differentiated macrophages were incubated in complete medium containing stationary phase *Leishmania* spp. promastigotes. After incubation, non-internalized promastigotes were removed and the infected macrophages were treated with the natural products. Parasite inhibition was therefore assessed either by microscopic counting using a compound or fluorescent microscope for GFP assay [51,59,60,76].

The resazurin/alamar blue, MTT and XTT assays consisted of incubating *Leishmania* parasites with plant products in microtiter plate format followed by addition of the dyes (resazurin or MTT or XTT) and further incubation for additional 2–3 h. In the case of the resazurin assay, the blue dye resazurin is reduced to the pink-coloured resorufin in the medium by cell activity (growing parasite); while for MTT and XTT, the yellow tetrazolium salt is reduced into blue-violet and orange formazan, respectively. These assays depend on an easily recognised colour change and parasite viability can be determined visually or measured spectrophotometrically [75,76,83].

Phosphatase activity is based on enzymatic hydrolysis of *p*-nitrophenyl phosphate followed by measurement of the incorporation of labeled nucleosides into nucleic acids. Phosphatase activity is proportional to the number of surviving parasites [84].

The in vivo assay involved the treatment of mice infected with promastigotes with various concentrations of drugs for a given period of time. Activity against visceral infection is assessed by measuring spleen or liver parasite burdens after giemsa-stained smear observation [76]. The antileishmanial drug screening reported mainly the use of parasite promastigotes and to a lesser extent, axenic amastigotes as they are easily maintained. The marine algae studied appeared to have good antileishmanial activity in vitro against the promastigote and axenic amastigote forms of *Leishmania* parasites (Table 1 and Table 2). Only 20 species (*Anadyomene saldanhae*, *Bryothamnion triquetrum*, *Canistrocarpus cervicornis*, *Caulerpa cupressoides*, *Ceramium nitens*, *Chondrococcus hornemanni*, *Cystoseira baccata*, *Dictyota mertensii*, *Dictyota pfaffii*, *Dictyota* sp., *Fucus vesiculosus*, *Halimeda opuntia*, *Lessonia vadosa*, *Laurencia aldingensis*, *Laurencia dendroidea*, *Ochtodes secundiramea*, *Padina* sp., *Palisada flagellifera*, *Palisada perforata* and *Stypopodium zonale*) were reportedly tested against the intracellular amastigote form. Extracts from some species such as *Dictyota* sp., *O. secundiramea* and *C. cupressoides* showed activity against *Leishmania* promastigotes, which is the insect vector-based form of the parasite. However, these extracts were inactive on intracellular amastigotes. In fact, the promastigote may not be the appropriate target for an antileishmanial drug due to significant cellular, physiological, biochemical and molecular differences when compared to intracellular amastigotes. Similarly, axenic amastigotes model has been developed to mimic the intracellular parasite stage; however it has been shown that some promising hits against this form of the parasite were inactive on intracellular amastigotes [85,86]. This limitation is due to several differences in cellular processes, including intracellular transport, response to oxidative stress, and metabolism. Moreover, with this model, the natural niche of the parasite, the host-parasite interactions, and the accessibility of the target have not been taken into account. Also, as evidenced in the reports, some marine algae extracts that showed activity against axenic amastigotes were inactive on the intracellular amastigote. This is likely due to their inability to cross host cells membrane or to maintain stability under low pH [87]. Fucoidan (12, Figure 1) was reported to be inactive on promastigotes of *L. donovani*, but it otherwise showed a good activity against the intracellular amastigote of the parasite [76]. Failure to identify all active compounds and selection of numerous false-positive hits has recently been associated with the use of the insect stage promastigotes and axenic amastigotes in primary screenings. Therefore, subsequent to using promastigotes or axenic amastigotes as models for screening, an important next step in the validation process should involve testing for activity on intracellular amastigotes that represents an appropriate target for an antileishmanial drug.

Microscopy counting method has been used for assaying drugs against intracellular amastigote form of *Leishmania* parasites (Table 1 and Table 2). Nevertheless, microscopic quantification of parasite burdens is laborious, time-consuming and requires specific processes including staining and microscopic observation. An alternative, the Trypanothione reductase (TryR)-based assay developed by Bogaart et al. [88] is a simple and efficient assay. It is a quantitative colorimetric assay in which the activity of a native enzyme (Trypanothione reductase) of the kinetoplast-unique thiol-redox metabolism is used to assess parasite viability by monitoring its 5,5′-dithiobis 2-nitrobenzoic acid-coupled reducing activity. More recently, another promising method was developed for *Leishmania* disease drug discovery called ex vivo model, which uses cell explants from infected rodents. This model involves real amastigote-infected organ macrophages with the full repertoire of immune cells that are important in both the pathogenesis of leishmaniasis and healing response to the disease. With ex vivo model, the replication of the intracellular amastigote could easily be quantified by measurement of luciferase activity within a system that mimics the immunopathological environment, which is known to strongly have an impact on parasite replication, killing, and drug efficacy [89,90].

To, date, only *Chondrococcus hornemanni*, *Fucus vesiculosus* and *Osmundaria obtusiloba* have been evaluated for antileishmanial activity using both the in vitro and in vivo models (Table 1 and Table 2). Extracts from *O. obtusiloba C. hornemanni* and fucoidan (12, Figure 1) isolated from *F. vesiculosus* showed promising in vitro antileishmanial activities that were confirmed in vivo [48,58,76]. In vitro assays play an essential role in drug discovery process because of their advantages consisting of a simplicity, convenience and short course, as well as limited amounts of samples used. However, most identified potent hits using in vitro assays do not translate their activities when tested in in vivo. In fact, in vivo assays provide an integrated system in which the efficacy of compound can be assessed in the physiological context [91,92] and can provide the combined effect of permeability, distribution, metabolism and excretion, yielding measurable sets of pharmacokinetic parameters and toxicology endpoints [91].

## 5. Conclusions

Given the shortcomings of existing treatments, there is an urgent need for novel drugs to treat *Leishmania* diseases. Marine algae have become an important base in research to discover new chemical entities with potential to be developed into drugs. In fact, the metabolic and physiological capabilities of marine algae that allow them to survive in a complex habitat provide a tremendous potential for the production of unique metabolites, which are not found in terrestrial environment. Recent trends in drug research from natural sources have indicated that marine algae are a promising source of novel active compounds, especially those with antileishmanial activity. Indeed, this review has documented the updated list of marine macroalgae and their isolated compounds that have been tested against *Leishmania* parasites. Compared to terrestrial plants, only few studies have been done with marine macroalgae and only 151 marine macroalgae were tested against *Leishmania* parasites highlighting a gap in knowledge and stressing the need for extensive attempts to systematically scrutinize these marine raw materials for new antileishmanial drugs. Species from Dictotaceae family and *Anadyomene*, *Laurencia* complex, *Ulva* and *Asparagopsis* genera are the most interesting macroalgae for antileishmanial drugs discovery. Moreover, analysis of the reports indicates that investigating marine macroalgal compounds has the potential as promising avenue for identifying novel compounds with potent antileishmanial activity and low toxicity. Such examples include elatol, obtusol, (4*R*,9*S*,14*S*)-4α-acetoxy-9β,14α-dihydroxydolast-1(15),7-diene and fucosterol that could be interesting scaffolds for the development of new and effective antileishmanial drugs. However, reports indicate that most marine algae have been tested against the promastigote and axenic amastigote forms of *Leishmania* parasites, and this might result in many false active natural products. This situation emphasizes the importance of using additional and sensitive advanced methods for drug discovery strategies against leishmaniasis including testing against the intracellular amastigotes, the relevant stage for pathogenesis of the disease. This strategy should avoid selection of fasely active samples or lack of detection of truely active samples and the efficacy study using animal models would be of great value for validation of the results.

Overall, the results reported till date have shown promising antileishmanial extracts/compounds from marine macroalgae that support further exploration for the discovery of new leads with high therapeutical value.

## Figures and Tables

**Table 1:** Antileishmanial activity of extracts from marine macroalgae.

Species of Marine Algae	Extraction Solvent	*Leishmania* Species	Parasites Stage	Method of Activity Evaluation	Activity (IC_50_ or % of Inhibition); Cytotoxicity (CC_50_)	References
**Phaeophyceae**						
*Ascophyllum nodosum* (Linnaeus) Le Jolis	Isopropyl alcohol- Chloroform/methanol extract	*L. donovani*	Axenic amastigote	In vitro: Resazurin assay	66.3 μg/mL(>90.0 μg/mL)	[40]
*Bifurcaria bifurcata* R. Ross	Isopropyl alcohol- Chloroform/methanol extract	*L. donovani*	Axenic amastigote	In vitro: Resazurin assay	6.4 μg/mL (32.7 μg/mL)	[40]
	Ethyl acetate extract	*L. donovani*	Axenic amastigote	In vitro: Resazurin assay	3.8 μg/mL (6.0 μg/mL)	[41]
	Hexane extract	*L. infantum*	Promastigote	In vitro: MTT assay	46.83 μg/mL (21.8 μg/mL)	[42]
	Ether extract	*L. infantum*	Promastigote	In vitro: MTT assay	51.64 μg/mL (40.46 μg/mL)	[42]
	Chloroform extract	*L. infantum*	Promastigote	In vitro: MTT assay	63.83 μg/mL (37.0 μg/mL)	[42]
*Canistrocarpus cervicornis* (Kützing) De Paula and De Clerck	Acetate extract	*L. amazonensis*	Promastigote	In vitro: Microscopic counting	50.0 μg/mL (50.0 μg/mL)	[43]
	Methanol extract	*L. amazonensis*	Promastigote	In vitro: Microscopic counting	100.0 μg/mL (51.0 μg/mL)	[43]
	Dichloromethane extract	*L. amazonensis*	Promastigote	In vitro: Microscopic counting	20.0 μg/mL (46.0 μg/mL)	[43]
	Ethyl acetate fraction	*L. amazonensis*	Promastigote	In vitro: Microscopic counting	8.0 μg/mL (47.0 μg/mL)	[43]
	Acetone extract	*L. braziliensis*	Promastigote	In vitro: Microscopic counting	85.8% inhibition at 50.0 μg/mL (>50.0 μg/mL)	[29]
*Chorda filum* (Linnaeus) Stackhouse	Isopropyl alcohol- Chloroform/methanol extract	*L. donovani*	Axenic amastigote	In vitro: Resazurin assay	21.1 μg/mL (>90.0 μg/mL)	[40]
*Colpomenia peregrina* Sauvageau	Isopropyl alcohol- Chloroform/methanol extract	*L. donovani*	Axenic amastigote	In vitro: Resazurin assay	29.1 μg/mL (>90.0 μg/mL)	[40]
*Cystoseira baccata* (S.G. Gmelin) P.C.	Isopropyl alcohol- Chloroform/methanol extract	*L. donovani*	Axenic amastigote	In vitro: Resazurin assay	15.7 μg/mL (>90.0 μg/mL)	[40]
	Hexane extract	*L. infantum*	Promastigote	In vitro: MTT assay	74.0% inhibition at 250.0 μg/mL (ND)	[44]
*Cystoseira barbata* (Stackhouse) C. Agardh	Methanol extract	*L. donovani*	Axenic amastigote	In vitro: Resazurin assay	23.46–69.98 μg/mL (>90.0 μg/mL)	[45]
*Cystoseira crinata* (Desf.) Duby	Methanol extract	*L. donovani*	Axenic amastigote	In vitro: Resazurin assay	28.21 μg/mL (>90.0 μg/mL)	[45]
*Cystoseira tamariscifolia* (Hudson) Papenf.	Isopropyl alcohol- Chloroform/methanol extract	*L. donovani*	Axenic amastigote	In vitro: Resazurin assay	19.6 μg/mL (62.5 μg/mL)	[40]
*Dictyopteris polypodioides* (A.P. de Candolle) J.V. Lamouroux	Ethyl acetate extract	*L. donovani*	Axenic amastigote	In vitro: Resazurin assay	10.8 μg/mL (87.0 μg/mL)	[41]
*Dictyota caribaea* Hörnig and Schnetter	Organic extract	*L. mexicana*	Promastigote	In vitro: Microscopic counting	24.4 μg/mL (≥1000.0 μg/mL)	[46]
*Dictyota ciliolata* Sonder ex Kützing	Hexane extract	*L. amazonensis*	Promastigote	In vitro: MTT assay	1.15 μg/mL (23.5 μg/mL)	[47]
	Ethyl acetate extract	*L. amazonensis*	Promastigote	In vitro: MTT assay	4.22 μg/mL (19.0 μg/mL)	[47]
*Dictyota dichotoma* (Hudson) J.V. Lamouroux	Ethanol extract	*L. donovani*	Axenic amastigote	In vitro: Resazurin assay	52.0 μg/mL (>90.0 μg/mL)	[48]
	Isopropyl alcohol- Chloroform/methanol extract	*L. donovani*	Axenic amastigote	In vitro: Resazurin assay	42.4 μg/mL (>90.0 μg/mL)	[40]
	Ethyl acetate extract	*L. donovani*	Axenic amastigote	In vitro: Resazurin assay	8.8 μg/mL (27.0 μg/mL)	[41]
*Dictyota menstrualis* (Hoyt) Schnetter, Hörning and Weber-Peuker	Ethyl acetate extract	*L. amazonensis*	Promastigote	In vitro: MTT assay	0.7–0.75 μg/mL (18.3–24.7 μg/mL)	[47]
	Hexane extract	*L. amazonensis*	Promastigote	In vitro: MTT assay	0.61 μg/mL (18.2 μg/mL)	[47]
*Dictyota mertensii* (Martius) Kützing	Dichloromethane/methanol extract	*L. amazonensis*	Promastigote	In vitro: Microscopic counting	71.60 μg/mL (233.10 μg/mL)	[49]
		*L. amazonensis*	Intracellular amastigote	In vitro: Microscopic counting	81.4 μg/mL (233.10 μg/mL)	[49]
*Dictyota* sp.	Dichloromethane/methanol extract	*L. braziliensis*	Promastigote	In vitro: MTT assay	93.3% inhibition at 50.0 μg/mL (>50.0 μg/mL)	[29]
*Fucus ceranoides* (Linnaeus)	Isopropyl alcohol- Chloroform/methanol extract	*L. donovani*	Axenic amastigote	In vitro: Resazurin assay	25.3 μg/mL (>90.0 μg/mL)	[40]
*Fucus serratus* (Linnaeus)	Isopropyl alcohol- Chloroform/methanol extract	*L. donovani*	Axenic amastigote	In vitro: Resazurin assay	34.1 μg/mL (>90.0 μg/mL)	[40]
*Fucus spiralis* (Linnaeus)	Isopropyl alcohol- Chloroform/methanol extract	*L. donovani*	Axenic amastigote	In vitro: Resazurin assay	34.3 μg/mL (>90.0 μg/mL)	[40]
*Fucus vesiculosus* (Linnaeus)	Isopropyl alcohol- Chloroform/methanol extract	*L. donovani*	Axenic amastigote	In vitro: Resazurin assay	33.0 μg/mL (>90.0 μg/mL)	[40]
*Halidrys siliquosa* (Linnaeus) Lyngb.	Isopropyl alcohol- Chloroform/methanol extract	*L. donovani*	Axenic amastigote	In vitro: Resazurin assay	8.6 μg/mL (45.0 μg/mL)	[40]
*Himanthalia elongata* (Linnaeus) S.F. Gray	Isopropyl alcohol- Chloroform/methanol extract	*L. donovani*	Axenic amastigote	In vitro: Resazurin assay	64.7 μg/mL (>90.0 μg/mL)	[40]
*Laminaria digitata* (Hudson) J.V. Lamouroux	Isopropyl alcohol- Chloroform/methanol extract	*L. donovani*	Axenic amastigote	In vitro: Resazurin assay	34.5 μg/mL (>90.0 μg/mL)	[40]
*Leathesia difformis* (Linnaeus) Aresch.	Isopropyl alcohol- Chloroform/methanol extract	*L. donovani*	Axenic amastigote	In vitro: Resazurin assay	77.4 μg/mL (>90.0 μg/mL)	[40]
*Lobophora variegata* (J.V. Lamouroux) Womersley ex E.C. Oliveira	Organic extracts	*L. mexicana*	Promastigote	In vitro: Microscopic counting	49.9 μg/mL (≥1000.0 μg/mL)	[46]
*Padina* sp.	Acetone extract	*L. braziliensis*	Promastigote	In vitro: MTT assay	80.9% inhibition at 50.0 μg/mL (300.4 μg/mL)	[29]
		*L. braziliensis*	Intracellular amastigote	In vitro: Microscopic counting	40.2 μg/mL (300.4 μg/mL)	[29]
*Pelvetia canaliculata* (Linnaeus) Decaisne and Thuret	Isopropyl alcohol- Chloroform/methanol extract	*L. donovani*	Axenic amastigote	In vitro: Resazurin assay	35.7 μg/mL (>90.0 μg/mL)	[40]
*Pylaiella littoralis* (Linnaeus) Kjellm.	Isopropyl alcohol- Chloroform/methanol extract	*L. donovani*	Axenic amastigote	In vitro: Resazurin assay	47.1 μg/mL (>90.0 μg/mL)	[40]
*Saccorhiza polyschides* (Lightf.) Batt.	Isopropyl alcohol- Chloroform/methanol extract	*L. donovani*	Axenic amastigote	In vitro: Resazurin assay	31.8 μg/mL (>90.0 μg/mL)	[40]
*Sargassum muticum* (Yendo) Fensholt	Polysaccharide extracts	*L. infantum*	Promastigote	In vitro: MTT assay	~98.5% inhibition at 160.0 μg/mL (ND)	[4]
	Isopropyl alcohol- Chloroform/methanol extract	*L. donovani*	Axenic amastigote	In vitro: Resazurin assay	34.7 μg/mL (>90.0 μg/mL)	[40]
*Sargassum natans* (Linnaeus) Gaill.	Ethanol extract	*L. donovani*	Axenic amastigote	In vitro: Resazurin assay	90.9 μg/mL (>90.0 μg/mL)	[48]
*Sargassum oligocystum* Montagne	Hot water extract	*L. major*	Promastigote	In vitro: MTT assay	78.0 μg/mL (ND)	[50]
*Scinaia furcellata* (Turn.) J. Agardh	Ethanol extract	*L. donovani*	Axenic amastigote	In vitro: Resazurin assay	64.4 μg/mL (>90.0 μg/mL)	[48]
*Scytosiphon lomentaria* (Lyngb.) Link	Isopropyl alcohol- Chloroform/methanol extract	*L. donovani*	Axenic amastigote	In vitro: Resazurin assay	34.3 μg/mL (>90.0 μg/mL)	[40]
*Stypocaulon scoparium* (L.) Kützing	Isopropyl alcohol- Trichloromethane/methanol extract	*L. donovani*	Axenic amastigote	In vitro: Resazurin assay	30.4 μg/mL (>90.0 μg/mL)	[40]
*Stypopodium zonale* (J.V. Lamouroux) Papenfuss	Dichloromethane extract	*L. amazonensis*	Promastigote	In vitro: Microscopic counting	100.0% inhibition at 10.0 μg/mL (>50.0 μg/mL)	[51]
		*L. amazonensis*	Intracellular amastigote	In vitro: Microscopic counting	0.27 μg/mL (>50.0 μg/mL)	[51]
	Ethyl acetate-hexane extract	*L. amazonensis*	Promastigote	In vitro: MTT assay	0.75 μg/mL (29.5 μg/mL)	[47]
*Turbinaria turbinata* (Linnaeus) Kuntze	Organic extracts	*L. mexicana*	Promastigote	In vitro: Microscopic counting	10.9 μg/mL (≥1000.0 μg/mL)	[46]
*Undaria pinnatifida* (Harvey) Suringar	Polysaccharide extracts	*L. infantum*	Promastigote	In vitro: MTT assay	~97.5% inhibition at 160.0 μg/mL (ND)	[4]
**Rhodophyta**						
*Asparagopsis armata* Harvey	Ethanol-hexane:ethyl acetate fraction	*L. donovani*	Promastigote	In vitro: Resazurin assay	10.0 μg/mL (ND)	[52]
	Ethanol-ethyl acetate fraction	*L. donovani*	Promastigote	In vitro: Resazurin assay	19.0 μg/mL (ND)	[52]
*Asparagopsis taxiformis* (Delile) Trevisan	Hexane extract	*L. donovani*	Promastigote	In vitro: Resazurin assay	17.0 μg/mL (ND)	[52]
	Dichloromethane extract	*L. donovani*	Promastigote	In vitro: Resazurin assay	16.0 μg/mL (ND)	[52]
	Ethanol-hexane:ethyl acetate fraction	*L. donovani*	Promastigote	In vitro: Resazurin assay	14.0 μg/mL (ND)	[52]
	Ethanol-ethyl acetate fraction	*L. donovani*	Promastigote	In vitro: Resazurin assay	20.0 μg/mL (ND)	[52]
	Ethanol extract	*L. infantum*	Promastigotes	In vitro: Microscopic counting	25.0 μg/mL (>90.0 μg/mL)	[53]
		*L. infantum*	Axenic amastigotes	In vitro: Microscopic counting	9.0 μg/mL (>90.0 μg/mL)	[53]
*Boergeseniella fruticulosa* (Wulfen) Kylin	Isopropyl alcohol- Chloroform/methanol extract	*L. donovani*	Axenic amastigote	In vitro: Resazurin assay	26.6 μg/mL (>90.0 μg/mL)	[54]
*Botryocladia leptopoda* (J.Agardh) Kylin	Ethanol extract	*L. major*	Promastigote	In vitro: Microscopic counting	60.81 μg/mL (ND)	[25,55]
*Bostrychia tenella* (J.V. Lamouroux) J. Agardh	Hexane fractions	*L. amazonensis*	Promastigote	In vitro: MTT assay	17.4 μg/mL (ND)	[56]
	Hexane subfractions (HO1; HO2; HO3; HO5; HO6)	*L. amazonensis*	Promastigote	In vitro: MTT assay	22.2; 1.5; 2.7; 31.7; 66.2 μg/mL (ND)	[56]
	Dichloromethane subfractions (DO1-DO2)	*L. amazonensis*	Promastigote	In vitro: MTT assay	4.4–4.3 μg/mL (ND)	[56]
*Bryothamnion triquetrum* (S.G. Gmelin) M. Howe	Aqueous extract	*L. amazonensis*	Promastigote	In vitro: Phosphatase assay	78.6 μg/mL (258.1 μg/mL)	[57]
*Calliblepharis jubata* (Goodenough and woodward) Kützing	Isopropyl alcohol- Chloroform/methanol extract	*L. donovani*	Axenic amastigote	In vitro: Resazurin assay	49.8 μg/mL (>90.0 μg/mL)	[54]
*Centroceras clavulatum* (C. Agardh) Montagne	Ethanol extract	*L. major*	Promastigote	In vitro: Microscopic counting	57.89–57.85 μg/mL (ND)	[25,55]
*Ceramium rubrum* (Hudson) C. Agardh	Methanol extract	*L. donovani*	Axenic amastigote	In vitro: Resazurin assay	16.76 μg/mL (>90.0 μg/mL)	[46]
*Ceramium virgatum* Roth	Isopropyl alcohol- Chloroform/methanol extract	*L. donovani*	Axenic amastigote	In vitro: Resazurin assay	25.6 μg/mL (>90.0 μg/mL)	[54]
*Chylocladia verticillata* (Lightf.) Bliding	Isopropyl alcohol- Chloroform/methanol extract	*L. donovani*	Axenic amastigote	In vitro: Resazurin assay	47.3 μg/mL (>90.0 μg/mL)	[54]
*Chondrococcus hornemanni* (Mert.) Schmitz	Ethanol extract	*L. donovani*	Promastigote	In vitro: Green fluorescent protein assay	29.5 μg/mL (>200.0 μg/mL)	[58]
		*L. donovani*	Intracellular amastigote	In vitro: Microscopic counting	40.6 μg/mL (>200.0 μg/mL)	[58]
		*L. donovani*		In vivo	75.38% suppression at 250.0 μg/mL	[58]
	*n*-butanol soluble fraction of ethanol extract	*L. donovani*	Promastigote	In vitro: Green fluorescent protein assay	54.2 μg/mL (>200.0 μg/mL)	[58]
		*L. donovani*	Intracellular amastigote	In vitro: Microscopic counting	61.0 μg/mL (>200.0 μg/mL)	[58]
	Chromatographic fraction of *n*-butanol soluble fraction (F011)	*L. donovani*	Promastigote	In vitro: Green fluorescent protein assay	53.7 μg/mL (>200.0 μg/mL)	[58]
		*L. donovani*	Intracellular amastigote	In vitro: Microscopic counting	57.4 μg/mL (>200.0 μg/mL)	[58]
		*L. donovani*		In vivo	76.2% suppression at 100.0 μg/mL	[58]
	Chromatographic fraction of *n*-butanol soluble fraction (F012)	*L. donovani*	Promastigote	In vitro: Green fluorescent protein assay	54.5 μg/mL (>200.0 μg/mL)	[58]
		*L. donovani*	Intracellular amastigote	In vitro: Microscopic counting	51.7 μg/mL (>200.0 μg/mL)	[58]
		*L. donovani*		In vivo	56.7% suppression at 100.0 μg/mL	[58]
*Chondrus crispus* Stackhouse	Ethanol extract	*L. donovani*	Axenic amastigote	In vitro: Resazurin assay	95.0% inhibition at 9.7 μg/mL (84.0 μg/mL)	[41]
*Claviclonium ovatum* (J.V. Lamouroux) Kraft and Min-Thein	Isopropyl alcohol- Chloroform/methanol extract	*L. donovani*	Axenic amastigote	In vitro: Resazurin assay	61.2 μg/mL (>90.0 μg/mL)	[54]
*Corallina officinalis* Linnaeus	Isopropyl alcohol- Chloroform/methanol extract	*L. donovani*	Axenic amastigote	In vitro: Resazurin assay	22.7 μg/mL (88.6 μg/mL)	[54]
*Corallina granifera* Ell. et Sol.	Methanol extract	*L. donovani*	Axenic amastigote	In vitro: Resazurin assay	35.02 μg/mL (>90.0 μg/mL)	[45]
*Cryptopleura ramosa* (Hudson) L. Newton	Isopropyl alcohol- Chloroform/methanol extract	*L. donovani*	Axenic amastigote	In vitro: Resazurin assay	85.6 μg/mL (>90.0 μg/mL)	[54]
*Cystoclonium purpureum* (Hudson) Batt.	Isopropyl alcohol- Chloroform/methanol extract	*L. donovani*	Axenic amastigote	In vitro: Resazurin assay	67.3 μg/mL (>90.0 μg/mL)	[54]
*Dasya pedicellata* (C. Agardh) C. Agardh	Methanol extract	*L. donovani*	Axenic amastigote	In vitro: Resazurin assay	23.04 μg/mL (14.7 μg/mL)	[45]
*Dilsea carnosa* (Schmidel) Kuntze	Ethyl acetate extract	*L. donovani*	Axenic amastigote	In vitro: Resazurin assay	9.5 μg/mL (74.0 μg/mL)	[41]
*Dumontia incrassata* (O.F. Müll.) J.V. Lamouroux	Isopropyl alcohol- Chloroform/methanol extract	*L. donovani*	Axenic amastigote	In vitro: Resazurin assay	68.6 μg/mL (>90.0 μg/mL)	[54]
*Furcellaria lumbricalis* (Hudson) J.V. Lamouroux	Isopropyl alcohol- Chloroform/methanol extract	*L. donovani*	Axenic amastigote	In vitro: Resazurin assay	43.3 μg/mL (>90.0 μg/mL)	[54]
*Gelidium crinale* (Hare ex Turner)	Methanol extract	*L. donovani*	Axenic amastigote	In vitro: Resazurin assay	19.95 μg/mL (>90.0 μg/mL)	[45]
*Gelidium pulchellum* (Turner) Kützing	Isopropyl alcohol- Chloroform/methanol extract	*L. donovani*	Axenic amastigote	In vitro: Resazurin assay	32.5 μg/mL (>90.0 μg/mL)	[54]
*Gracilaria bursa-pastoris* (S.G. Gmelin) P.C. Silva	Polysaccharide extracts	*L. infantum*	Promastigote	In vitro: MTT assay	~87.5.0% inhibition at 160.0 μg/mL (ND)	[4]
*Gracilaria corticata* (J. Agardh) J. Agardh	Ethanol extract	*L. major*	Promastigote	In vitro: Microscopic counting	37.5 μg/mL (ND)	[25,55]
	Cold water extract	*L. major*	Promastigote	In vitro: MTT assay	65.0 μg/mL (ND)	[50]
	Hot water extract	*L. major*	Promastigote	In vitro: MTT assay	38.0 μg/mL (ND)	[50]
*Gracilaria gracilis* (Stackhouse) Steentoft, L.M. Irvine and Farnham	Isopropyl alcohol- Chloroform/methanol extract	*L. donovani*	Axenic amastigote	In vitro: Resazurin assay	53.3 μg/mL (>90.0 μg/mL)	[54]
*Gracilaria salicornia* (C. Agardh) E.Y. Dawson	Cold water extract	*L. major*	Promastigote	In vitro: MTT assay	74.0 μg/mL (ND)	[50]
	Hot water extract	*L. major*	Promastigote	In vitro: MTT assay	46.0 μg/mL (ND)	[50]
*Gracilaria verrucosa* (Hudson) Papenfuss	Methanol extract	*L. donovani*	Axenic amastigotes	In vitro: Resazurin assay	36.02 μg/mL (>90.0 μg/mL)	[45]
*Gracilaria viridis* Sfriso, Wolf, Sciuto, Morabito, Andreoli and Moro	Polysaccharide extracts	*L. infantum*	Promastigote	In vitro: MTT assay	~82.8% inhibition at 160.0 μg/mL (ND)	[4]
*Halopitys incurvus* (Hudson) Batt.	Isopropyl alcohol- Chloroform/methanol extract	*L. donovani*	Axenic amastigote	In vitro: Resazurin assay	16.5 μg/mL (>90.0 μg/mL)	[54]
*Halurus equisetifolius* (Lightf.) Kützing	Isopropyl alcohol- Chloroform/methanol extract	*L. donovani*	Axenic amastigote	In vitro: Resazurin assay	69.2 μg/mL (>90.0 μg/mL)	[54]
*Jania rubens* (Linnaeus) J.V. Lamouroux	Isopropyl alcohol- Chloroform/methanol extract	*L. donovani*	Axenic amastigote	In vitro: Resazurin assay	60.7 μg/mL (>90.0 μg/mL)	[54]
	Methanol extract	*L. donovani*	Axenic amastigote	In vitro: Resazurin assay	28.0 μg/mL (>90.0 μg/mL)	[45]
*Laurencia aldingensis* Saito and Womersley	Dichloromethane/methanol extract	*L. amazonensis*	Promastigote	In vitro: Green fluorescent protein assay	24.5 μg/mL (259.8 μg/mL)	[59]
		*L. amazonensis*	Intracellular amastigote	In vitro: Microscopic counting	12.5 μg/mL (259.8 μg/mL)	[59]
*Laurencia dendroidea* (Hudson) J.V. Lamouroux	Lipophilic extracts	*L. amazonensis*	Promastigote	In vitro: Green fluorescent protein assay	17.9–34.2 μg/mL (106.2–131.7 μg/mL)	[60]
		*L. amazonensis*	Intracellular amastigote	In vitro: Microscopic counting	8.7–10.8 μg/mL (106.2–131.7 μg/mL)	[60]
	Dichloromethane/methanol extract	*L. amazonensis*	Promastigote	In vitro: Green fluorescent protein assay	30.1–97.2 μg/mL (187.0–240.0 μg/mL)	[60]
		*L. amazonensis*	Intracellular amastigote	In vitro: Microscopic counting	16.8–22.4 μg/mL (187.0–240.0 μg/mL)	[60]
*Laurencia microcladia* Kützing	Organic extracts	*L. mexicana*	Promastigote	In vitro: Microscopic counting	16.3 μg/mL (119.8 μg/mL)	[46]
*Lomentaria articulata* (Hudson) Lyngb	Isopropyl alcohol- Chloroform/methanol extract	*L. donovani*	Axenic amastigote	In vitro: Resazurin assay	60.0 μg/mL (>90.0 μg/mL)	[54]
*Mastocarpus stellatus* (Stackhouse) Guiry	Isopropyl alcohol- Trichloromethane/methanol extract	*L. donovani*	Axenic amastigote	In vitro: Resazurin assay	44.1 μg/mL (>90.0 μg/mL)	[54]
*Melanothamnus afaqhusainii* M. Shameel	Ethanol extract	*L. major*	Promastigote	In vitro: Microscopic counting	32.6–32.5 μg/mL (ND)	[25,55]
*Ochtodes secundiramea* (Montagne) M. Howe	Acetone extract	*L. braziliensis*	Promastigote	In vitro: MTT assay	99.7% inhibition at 50.0 μg/mL (>50.0 μg/mL)	[29]
*Osmundaria obtusiloba* (C. Agardh) R.E. Norris	Ethanol extract	*L. amazonensis*	Promastigote	In vitro: MTT assay	24.5 μg/mL (240.0 μg/mL)	[47]
		*L. amazonensis*		In vivo	Active at 5.0 and 20.0 mg/kg	[47]
	Ethyl acetate-Hexane extract	*L. amazonensis*	Promastigote	In vitro: MTT assay	22.0 μg/mL (198.0 μg/mL)	[47]
*Osmundea hybrida* (A.P. de Candolle) K.W. Nam	Isopropyl alcohol- Chloroform/methanol extract	*L. donovani*	Axenic amastigote	In vitro: Resazurin assay	49.2 μg/mL (>90.0 μg/mL)	[54]
*Osmundea pinnatifida* (Hudson) Stackhouse	Ethanol extract	*L. major*	Promastigote	In vitro: Microscopic counting	6.25 μg/mL (ND)	[55]
	Isopropyl alcohol- Chloroform/methanol extract	*L. donovani*	Axenic amastigote	In vitro: Resazurin assay	32.7 μg/mL (>90.0 μg/mL)	[54]
*Palisada flagellifera* (J. Agardh) K.W. Nam	Dichloromethane/methanol extract	*L. amazonensis*	Promastigote	In vitro: Green fluorescent protein assay	30.7 μg/mL (198.0 μg/mL)	[59]
		*L. amazonensis*	Intracellular amastigote	In vitro: Microscopic counting	34.5 μg/mL (198.0 μg/mL)	[59]
*Palisada perforata* (Bory) K.W. Nam	Dichloromethane/methanol extract	*L. amazonensis*	Promastigote	In vitro: Green fluorescent protein assay	36.1–46.7 μg/mL (267.0 μg/mL)	[59]
		*L. amazonensis*	Intracellular amastigote	In vitro: Microscopic counting	29.7–34.5 μg/mL (267.0 μg/mL)	[59]
*Plocamium cartilagineum* (Linnaeus) P.S. Dixon	Isopropyl alcohol- Chloroform/methanol extract	*L. donovani*	Axenic amastigote	In vitro: Resazurin assay	21.2 μg/mL (>90.0 μg/mL)	[54]
*Polyides rotundus* (Hudson) Gaillon	Isopropyl alcohol- Chloroform/methanol extract	*L. donovani*	Axenic amastigote	In vitro: Resazurin assay	57.3 μg/mL (>90.0 μg/mL)	[54]
*Porphyra linearis* Grev	Isopropyl alcohol- Chloroform/methanol extract	*L. donovani*	Axenic amastigote	In vitro: Resazurin assay	55.5 μg/mL (>90.0 μg/mL)	[54]
*Scinaia Fascicularis* (Børgesen) Huisman	Ethanol extract	*L. major*	Promastigote	In vitro: Microscopic counting	59.6 μg/mL (ND)	[55]
*Scinaia hatei* Børgesen	Ethanol extract	*L. major*	Promastigote	In vitro: Microscopic counting	14.1 μg/mL (ND)	[25,55]
*Scinaia indica* Børgesen	Ethanol extract	*L. major*	Promastigote	In vitro: Microscopic counting	59.6 μg/mL (ND)	[25]
**Chlorophyceae**						
*Anadyomene saldanhae* A.B. Joly and E.C. Oliveira	Acetone extract	*L. braziliensis*	Promastigote	In vitro: MTT assay	87.9% inhibition at 50.0 μg/mL (294.2 μg/mL)	[29]
		*L. braziliensis*	Intracellular amastigote	In vitro: Microscopic counting	23.9 μg/mL (294.2 μg/mL)	[29]
*Caulerpa racemosa* (Forsskål) J. Agardh	Methanol extract	*L. donovani*	Axenic amastigote	In vitro: Resazurin assay	22.66 μg/mL (>90.0 μg/mL)	[45]
	Ethyl acetate-Hexane extract	*L. amazonensis*	Promastigote	In vitro: MTT assay	47.5–154 μg/mL (48.0–115.0 μg/mL)	[47]
*Caulerpa cupressoides* (Vahl) C. Agardh	Acetone extract	*L. braziliensis*	Promastigote	In vitro: MTT assay	51.7% inhibition at 50.0 μg/mL (>50.0 μg/mL)	[29]
*Caulerpa sertularioides* (S.G. Gmelin) M. Howe	Hot water extract	*L. major*	Promastigote	In vitro: MTT assay	85.0 μg/mL (ND)	[50]
*Cladophora rupestris* (L.) Kützing	Isopropyl alcohol- Chloroform/methanol extract	*L. donovani*	Axenic amastigote	In vitro: Resazurin assay	20.2 μg/mL (>90.0 μg/mL)	[61]
*Codium bursa* (L.) C. Agardh	Methanol extract	*L. donovani*	Axenic amastigote	In vitro: Resazurin assay	31.71 μg/mL (>90.0 μg/mL)	[45]
*Codium fragile* (Sur.) Hariot *ssp. Tomentosoides* (van Goor) Silva	Isopropyl alcohol- Chloroform/methanol extract	*L. donovani*	Axenic amastigote	In vitro: Resazurin assay	16.6 μg/mL (>90.0 μg/mL)	[61]
*Halimeda opuntia* (L.) Lamouroux	Aqueous	*L. amazonensis*	Promastigote	In vitro: Phosphatase assay	83.5 μg/mL (526.4 μg/mL)	[57]
		*L. amazonensis*	Intracellular amastigote	In vitro: Microscopic counting	70.7 μg/mL (526.4 μg/mL)	[57]
*Ulva intestinalis* Linnaeus	Isopropyl alcohol- Chloroform/methanol extract	*L. donovani*	Axenic amastigote	In vitro: Resazurin assay	14.9 μg/mL (>90.0 μg/mL)	[61]
*Ulva lactuca* Linnaeus	Ethanol extract	*L. donovani*	Axenic amastigote	In vitro: Resazurin assay	5.9 μg/mL (>90.0 μg/mL)	[48]
	Isopropyl alcohol- Chloroform/methanol extract	*L. donovani*	Axenic amastigote	In vitro: Resazurin assay	12.0 μg/mL (>90.0 μg/mL)	[61]

**Table 2:** Overview of the current status on natural products from marine macroalgae with antileishmanial potency.

Chemical Type	Chemical Classe	Isolated Compounds	Species of Marine Algae; Phylum	*Leishmania* Species	Parasites Stage	Method of Activity Evaluation	Activity (IC_50_ or % of Inhibition) Cytotoxicity (CC_50_)	References
Diterpene	Dolastane	(4*R*,9*S*,14*S*)-4α-acetoxy-9β, 14α-dihydroxydolast-1(15), 7-diene	*Canistrocarpus cervicornis* (Kützing) De Paula and De Clerck; Phaeophyceae	*L. amazonensis*	Promastigote	In vitro: Microscopic counting	2.0 μg/mL (186.0 μg/mL)	[43]
				*L. amazonensis*	Axenic amastigote	In vitro: Microscopic counting	12.0 μg/mL (186.0 μg/mL)	[43]
				*L. amazonensis*	Intracellular amastigote	In vitro: Microscopic counting	4.0 μg/mL (186.0 μg/mL)	[43]
	Meroditerpenoid	(3*R*)- and (3*S*)-tetraprenyltoluquinol (1a/1b) (3*R*)- and (3*S*)-tetraprenyltoluquinone (2a/2b)	*Cystoseira baccata* (S.G. Gmelin) P.C.; Phaeophyceae	*L. infantum*	Promastigote	In vitro: MTT assay	44.9 μM (126.6 μM)	[44]
				*L. infantum*	Intracellular amastigote	In vitro: MTT assay	25.0 μM (126.6 μM)	[44]
				*L. infantum*	Promastigote	In vitro: MTT assay	94.4 μM (84.5 μM)	[44]
		Atomaric acid	*Stypopodium zonale* (J.V. Lamouroux) Papenfuss; Phaeophyceae	*L. amazonensis*	Promastigote	In vitro: Microscopic counting	86.0% inhibition at 50.0 μM (169.5 μM)	[51]
				*L. amazonensis*	Intracellular amastigote	In vitro: Microscopic counting	20.2 μM (169.5 μM)	[51]
	Hydroazulene	Mixture of isomers pachydictyol A/isopachydictyol A	*Dictyota menstrualis* (Hoyt) Schnetter, Hörning and Weber-Peuker; Phaeophyceae	*L. amazonensis*	Promastigote	In vitro: MTT assay	23.5 μg/mL (30.0 μg/mL)	[47]
	Dolabellane	Dolabelladienetriol	*Dictyota pfaffii* Schnetter; Phaeophyceae	*L. amazonensis*	Promastigote	In vitro: Microscopic counting	95.5% Inhibition at 100.0 μM (>100.0 μM)	[14]
				*L. amazonensis*	Intracellular amastigote	In vitro: Microscopic counting	43.9 μM (>100.0 μM)	[14]
				*Leishmania/HIV-1* co-infection	Intracellular amastigote/HIV-1	In vitro: Microscopic counting	56.0% inhibition at 50.0 μM (>100.0 μM)	[14]
	Linear diterpene	Bifurcatriol	*Bifurcaria bifurcata* R.Ross; Phaeophyceae	*L. donovani*	Axenic amastigote	In vitro: Resazurin assay	18.8 μg/mL (56.6 μg/mL)	[74]
Triterpene	Phytosterol	Fucosterol	*Lessonia vadosa* Searles; Phaeophyceae	*L. infantum*	Promastigote	In vitro: Resazurin assay	45.0 μM (>100.0 μM)	[75]
				*L. infantum*	Intracellular amastigote	In vitro: Resazurin assay	10.30 μΜ (>100.0 μM)	[75]
				*L. amazonensis*	Promastigote	In vitro: Resazurin assay	55.0 μM (>100.0 μM)	[75]
				*L. amazonensis*	Intracellular amastigote	In vitro: Resazurin assay	7.89 μM (>100.0 μM)	[75]
Sesquiterpene	Chamigrene	Elatol	*Laurencia dendroidea* (Hudson) J.V. Lamouroux; Rhodophyta	*L. amazonensis*	Promastigote	In vitro: Microscopic counting and Green fluorescent protein assay	4.0 μM; 9.7 μg/mL (1.4 μM; 112.9–120.0 μg/mL)	[26,60]
				*L. amazonensis*	Intracellular amastigote	In vitro: Microscopic counting	0.45 μM; 4.5 μg/mL (1.4 μM; 112.9–120.0 μg/mL)	[26,60]
		Obtusol		*L. amazonensis*	Promastigote	In vitro: Green fluorescent protein assay	6.2 μg/mL (133.5–139.3 μg/mL)	[60]
				*L. amazonensis*	Intracellular amastigote	In vitro: Microscopic counting	3.9 μg/mL (133.5–139.3 μg/mL)	[60]
	Triquinane	Silphiperfol-5-en-3-ol		*L. amazonensis*	Promastigote	In vitro: Green fluorescent protein assay	43.8 μg/mL (160.2–172.8 μg/mL)	[60]
				*L. amazonensis*	Intracellular amastigote	In vitro: Microscopic counting	48.7 μg/mL (160.2–172.8 μg/mL)	[60]
Sulfated polysaccharide	Fucan	Fucoidan	*Fucus vesiculosus* (Linnaeus); Phaeophyceae	*L. donovani*	Intracellular amastigote	In vitro: Microscopic counting	93.0% inhibition at 50.0 μg/mL	[76]
				*L. donovani*		In vivo	100.0% supression at 200.0 mg/kg/day (ND)	[76]
	ND	Sulfated polysaccharide (NI)	*Botryoclada occidentalis* (Børgesen) Kylin; Rhodophyta	*L. amazonensis*	Promastigote	In vitro: MTT assay	63.7 μg/mL (27.3 μg/mL)	[77]
			*Caulerpa racemosa* (Forsskål) J. Agardh; Chlorophyta	*L. amazonensis*	Promastigote	In vitro: MTT assay	34.5 μg/mL (49.3 μg/mL)	[77]

IC_50_: Compound concentration that inhibited the proliferation of parasites by 50%; (CC_50_): Compound concentration that inhibited the proliferation of normal mammalian cells by 50%; ND: not determined; NI: Not identified.
